# Impact of adolescent complex regional pain syndrome on the psychopathology of young men ahead of military service: a retrospective cohort analysis of Korean conscription data

**DOI:** 10.1186/s40779-020-00294-0

**Published:** 2020-12-21

**Authors:** Shin-Heon Lee, Myeong-Jin Ko, Taek-Kyun Nam, Jeong-Taik Kwon, Yong-Sook Park

**Affiliations:** Department of Neurosurgery, College of Medicine, Chung-Ang University, Seoul, 06973 South Korea

**Keywords:** Chronic pain, Complex regional pain syndromes, Psychopathology, Personality inventory, Adolescent, Military personnel

## Abstract

**Background:**

The relationship between physical and psychopathological features in complex regional pain syndrome (CRPS) has been a subject of constant interest, but no data are available in adolescents. Therefore, we aimed to identify the factors associated with psychopathology in adolescents with CRPS ahead of military service.

**Methods:**

We retrospectively reviewed all conscription examinees who had completed a Military Personality Inventory (MPI) during a period between February 2013 and December 2016. A total of 63 persons with a history of CRPS (19-years of age for all) were enrolled. Basic demographic and pain-related data were analyzed to examine their association with MPI results. The mean FGR score as well as the 8 subdomain scores were compared between those with pain duration at < 15 months (*n* = 30) versus ≥15 months (*n* = 33). Binary MPI results (normal-abnormal) were also compared between the two groups.

**Results:**

In multivariate analysis, abnormal MPI was associated with pain duration, with an odds ratio (*OR*) at 1.05 for every 1-month increase (95% confidence interval (CI) 1.02–1.08; *P* = 0.002). Subjects with pain duration at ≥15 months have lower faking good response score (*P* < 0.001 vs. those with pain duration at < 15 months), and higher abnormal MPI result rate, faking bad response, inconsistency, anxiety, depression, somatization, paranoid, personality disorder cluster A, and personality disorder cluster B scores (*P* < 0.05). Pain duration was significantly associated with the MPI variables.

**Conclusions:**

Pain duration is associated with psychopathology in adolescents with CRPS. Psychopathologic features increased as the disease duration increased. A comprehensive understanding of time-dependent psychopathological factors could support the planning of multimodal approaches for managing adolescent CRPS.

## Background

Complex regional pain syndrome (CRPS) is a severe pain disorder that affects one or more extremities and typically develops after a trauma or nerve lesions. It is clinically characterized by sensory, autonomic, motor, and trophic symptoms. Causes of CRPS include autoimmune response, abnormal cytokine production, inflammatory processes, sympathetic-sensory disorders, and functional changes in the central nervous system [1].

The perspective that psychological factors contribute to the development of the disease has been the topic of constant controversy [2–4]. Nonetheless, researchers and clinicians agree on the reciprocal relationship between the physical and psychological components of pain [5]. Although it has been continuously debated whether these symptoms are the result or the cause of this debilitating disease, it is clear that CRPS is associated with psychological sequelae, such as depression, anxiety, poor quality of life, and functional disabilities [5, 6]. Despite these profound associations, previous studies have only focused on the presence of CRPS and psychopathology but have not separately investigated the pain-related factors and the demographic (and other) factors that potentially affect psychopathology.

CRPS could occur in both adults and adolescents [7]. Adolescent CRPS typically starts at 12–13 years, with a mean time from initial signs to diagnosis at 12 months [7, 8]. Compared with adult CRPS, adolescent CRPS has a relatively mild course and responds well to treatment [9]. Despite the relatively benign disease course, the risk of CRPS-induced psychopathology is a major challenge. Elevated risks of somatization, anxiety, and depression have been identified among patients with adolescent or pediatric CRPS [10]. Additionally, pain duration has been associated with suicidal ideation, and this association is mediated by depression among patients with “amplified pain”, the spectrum of which includes CRPS [11]. Considering the developmental and social aspects relevant to adolescent mental health, the psychopathology associated with adolescent CRPS is an important problem worthy of investigation and intervention.

In this study, we attempted to identify the factors that are associated with psychopathology among adolescents with CRPS ahead of military service.

## Methods

### Patients

This is a registry-based retrospective cohort study. All conscription examinees during a period between February 2013 and December 2016 were screened. Among the 1,418,519 19-year-old examinees evaluated for Korean conscription from February 2013 through December 2016, 72 self-reported medical histories of CRPS. We excluded seven conscripts who had a medical history of other psychiatric disorders before the diagnosis of CRPS and two conscripts whose pain symptoms did not correspond with the International Association for the Study of Pain (IASP) Budapest Criteria for CRPS according to the findings of physical examination. The final analysis included 63 subjects. The study received ethical approval from the Korean Military Manpower Administration. Anonymity was maintained throughout the study. Informed consent was not applicable.

### Demographic & disease characteristics

CRPS symptoms and signs were primarily documented as per medical records and verified by doctors during the overall pre-conscription physical assessments. CRPS type, site, symptom, intensity, and duration of the pain, as well as the treatment (physical or pharmacological therapy, sympathetic nerve intervention, or spinal cord stimulation), were recorded. Demographic characteristics in the analysis included age, sex, education, socioeconomic status, and parental coresidence status.

The IASP Budapest Criteria [12] were used to assess self-reported symptoms as well as signs observed during physical examinations. Harden et al. [13] developed the CRPS Severity Score (CSS), which evaluates the severity of CRPS on a 17-point scale (symptoms, 8 points; signs, 9 points). This scoring system corresponds well with the ISAP Budapest Criteria but provides assessment with a continuous scale [13, 14]. In this study, a modified CRPS severity score (mCSS) system that consisted of signs only (maximum score: 9 points) was used. The pain was assessed using an 11-point numerical rating scale (NRS; lowest score: 0; highest score: 10) [15].

### Psychopathologic assessment

The Military Personality Inventory (MPI) [16] was used to evaluate personality structure and psychopathology [16]. The MPI is a multiphasic personality inventory developed by the Korea Institute for Defense Analysis (KIDA) in 2006 and is based on the Minnesota Multiphasic Personality Inventory (MMPI) [17, 18]. It consists of 238 questions divided into four sections. The results are expressed as standardized T-scores [17, 18]. The overall MPI result was classified as “normal” when the results of the combined MPI scales met the conscription criteria, and MPI results were classified as “abnormal” when this condition was not satisfied [17, 18].

The validity and reliability of the MPI have been established by previous research [19–21]. In a feasibility study, MPI scores differed significantly between the maladjusted and psychotic group and the healthy controls [22]. The test-retest reliability was 0.69 (95% confidence interval [CI], 0.63–0.75), and Cronbach’s alpha was 0.72 (95% CI, 0.67–0.76) [23].

Details of MPI have been described previously [24]. Briefly, MPI is based on the results of the four sections (validity, neurosis, psychosis, and personality disorders). The validity section consists of four scales: faking good response (FGR), faking bad response (FBR), infrequency (INF), and inconsistency (INC). The neurosis section consists of anxiety (ANX), depression (DEP), and somatization (SOM) scales. For each of these scales, a higher score indicates a greater tendency to manifest mental illness symptoms. The psychosis section consists of schizophrenia (SCZ) and paranoid (PAR) scales. The personality disorder section consists of personality disorder cluster A (PDA), which comprises paranoid personality disorder, schizoid personality disorder, and schizotypal personality disorder, and personality disorder cluster B (PDB), which comprises antisocial personality disorder, borderline personality disorder, histrionic personality disorder, and narcissistic personality disorder [24].

We examined both the scores using the above-mentioned scales and the overall rate of “normal” versus “abnormal”. Subjects with pain duration at < 15 months (*n* = 30) versus ≥15 months (*n* = 33) were compared.

### Statistical analysis

SPSS Statistics for Windows, version 22 (IBM Corp., Armonk, New York, USA) and MedCalc, version 19.0.7 (MedCalc Software, Mariakerke, Belgium) were used for all statistical analyses. Categorical variables are expressed as frequencies or percentages and analyzed using the chi-square test or Fisher’s exact test. The factors associated with abnormal MPI results were analyzed using multivariate logistic regression. Receiver operating characteristic (ROC) curve analysis was performed to estimate the predictive accuracy of pain duration for the binary (normal-abnormal) MPI results. The cutoff values were selected to maximize the sum of sensitivity and specificity. Continuous variables with normal distribution were analyzed using Student’s *t*-test. Continuous variables not following normal distribution were analyzed using the Mann-Whitney *U* test. To analyze the impact of each clinical measurement on the MPI scores, linear regression analysis was performed. Multivariable linear regression analysis was performed to analyze the combined effect of the clinical measurements on MPI scores. *P* values < 0.05 were considered statistically significant. For multiple comparisons, the Bonferroni correction was conducted.

## Results

### Patient characteristics

Sixty-three male subjects with CRPS had a median age of 19.3 years and mean pain duration of 27.41 ± 2.67 months. Of the 63 adolescents with CRPS, 20 (31.7%) had abnormal MPI results. Forty-nine subjects were diagnosed as type I CRPS (77.8%) and 14 were diagnosed as type II CRPS. Demographic and pain characteristics including education, socioeconomic status, parental coresidence status, mCSS, NRS score, affected site, symptomatic presentation, and treatment modality, are shown in Table 1.

**Table 1:** Basic demographics and pain characteristics of adolescents with complex regional pain syndrome (CRPS)

Characteristic	Value
Age [year, median (range)]	19.3 (19–20)
Male [*n* (%)]	63 (100)
Education [*n* (%)]	
College or more	40 (63.5)
High school or less	23 (36.5)
Socioeconomic status [*n* (%)]	
High	8 (12.7)
Middle	33 (52.4)
Low	22 (34.9)
Parental coresidence status [*n* (%)]	
Both	54 (85.7)
One parent	8 (12.7)
None	1 (1.6)
Abnormal MPI results [*n* (%)]	20 (31.7)
mCSS (2–9, *x* ± *s*)	4.05 ± 1.58
NRS (0–10*, x ± s*)	6.25 ± 1.57
Pain duration (month*, x ± s*)	27.41 ± 2.67
CRPS type [*n* (%)]	
I	49 (77.8)
II	14 (22.2)
Affected site [*n* (%)]	
Upper extremity	18 (28.6)
Lower extremity	43 (68.3)
Multiple	2 (3.2)
Symptomatic presentation [*n* (%)]	
Allodynia	45 (71.4)
Hyperalgesia	42 (66.7)
Temperature asymmetry	34 (54.0)
Skin color asymmetry	36 (57.1)
Sweating asymmetry	28 (44.4)
Asymmetric edema	26 (41.3)
Trophic changes	12 (19.0)
Motor dysfunction	21 (33.3)
Decreased active range of motion	8 (12.7)
Treatment [*n* (%)]	
Physical or pharmacological therapy	21 (33.3)
Sympathetic nerve intervention	33 (52.4)
Spinal cord stimulation	9 (14.3)

*MPI* Military Personality Inventory, *mCSS* Modified Complex regional pain syndrome Severity Score, *NRS* Numerical rating scale, *CRPS* Complex regional pain syndrome

### Factors associated with abnormal MPI results

In the multivariate logistic regression analysis, abnormal MPI results were associated with pain duration, with an odds ratio (*OR*) at 1.05 with every 1-month increase (95% CI, 1.02–1.08; *P* = 0.002, Table 2). No other factors were associated with abnormal MPI.

**Table 2:** Influence of demographics and pain characteristics on Military Personality Inventory (MPI) results of adolescents with complex regional pain syndrome (CRPS)

Variable	Prevalence		Logistic regression analysis		
	Normal MPI (*n* = 43)	Abnormal MPI (*n* = 20)	*OR*	95% CI	*P*
Age (year, median)	19.35	19.28	0.25	0.01–4.24	0.334
Education [*n* (%)]					
College or more	28 (65.1)	12 (60.0)	1		
High school or less	15 (34.9)	8 (40.0)	0.99	0.22–4.52	0.986
Socioeconomic status [*n* (%)]					
High	7 (16.3)	1 (5.0)	1		
Middle	23 (53.5)	10 (50.0)	14.38	0.50–413.72	0.120
Low	13 (30.2)	9 (45.0)	23.71	0.81–695.42	0.066
Parental coresidence status [*n* (%)]					
Both	36 (83.7)	18 (90.0)	1		
One parent	6 (14.0)	2 (10.0)	0.41	0.02–7.00	0.536
None	1 (2.3)	0 (0.0)	0	0	1.000
mCSS (2–9)	4.21	3.70	0.73	0.37–1.43	0.358
NRS (0–10)	6.40	5.95	0.97	0.56–1.68	0.358
Pain duration (month)	21.40	40.35	1.05	1.02–1.08	0.002*
CRPS type [*n* (%)]					
I	34 (79.1)	15 (75.0)	1		
II	9 (20.9)	5 (25.0)	3.76	0.61–23.33	0.155
Affected site [*n* (%)]					
Upper extremity	12 (27.9)	6 (30.0)	1		
Lower extremity	29 (67.4)	14 (70.0)	0.98	0.20–4.90	0.983
Multiple	2 (4.7)	0 (0.0)	0	0	0.999
Treatment [*n* (%)]					
Physical or pharmacological therapy	15 (34.9)	6 (30.0)	1		
Sympathetic nerve intervention	22 (51.2)	11 (55.0)	6.90	0.76–62.51	0.086
Spinal cord stimulation	6 (14.0)	3 (15.0)	4.06	0.38–43.02	0.245

*MPI* Military Personality Inventory, *OR* Odds ratio, *CI* Confidence interval, *mCSS* Modified Complex regional pain syndrome Severity Score, *NRS* Numerical rating scale, *CRPS* Complex regional pain syndrome

### Prediction of MPI outcomes with pain duration

**Fig. 1:**
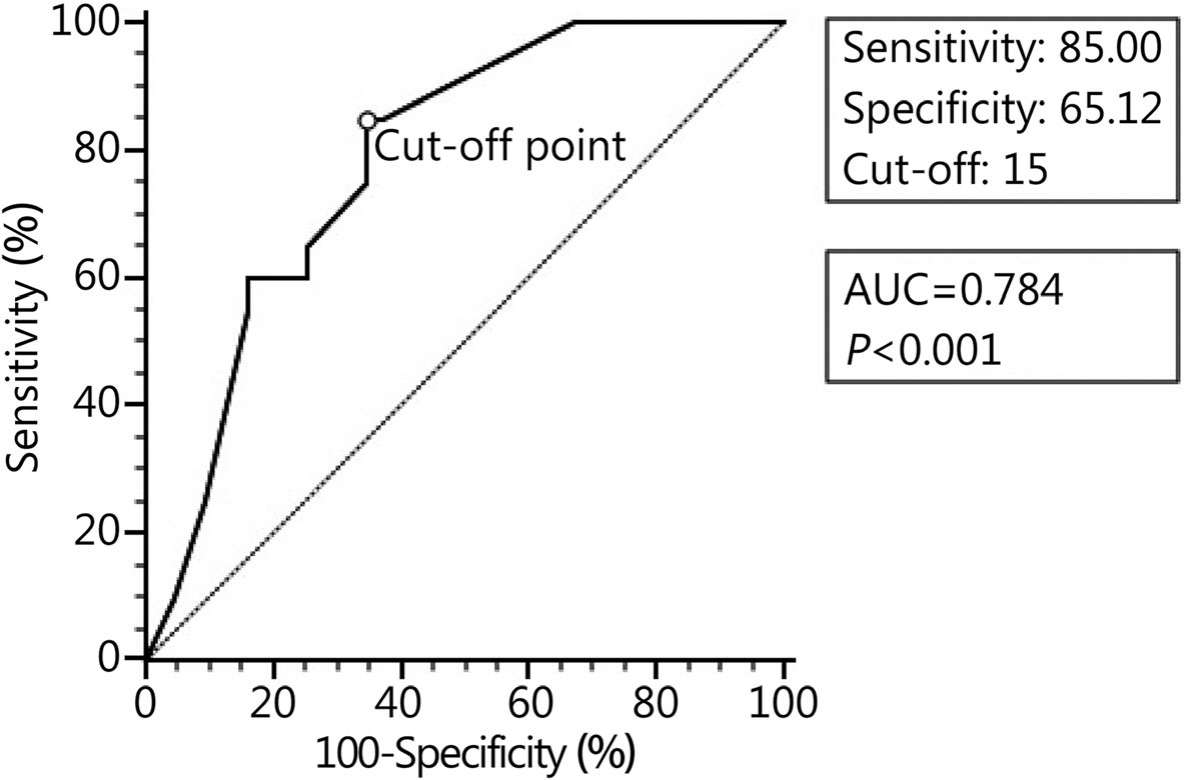
Receiver operating characteristic (ROC) curves using pain duration as a predictor and Military Personality Inventory (MPI) results (normal vs. abnormal) as a binary outcome

At a cutoff value at 15 months, pain duration predicted abnormal MPI with the area under the curve (AUC) in the ROC analysis at 0.784 (95% CI, 0.663–0.878; *P* < 0.001). The sensitivity and specificity were 85.0 and 65.1%, respectively (Fig. 1).

### Comparison of those with pain duration at < 15 vs. ≥15 months

There were no significant differences between subjects with pain duration at < 15 vs. ≥15 months in age, education, socioeconomic status, parental coresidence status, mean mCSS, mean NRS score, CRPS type, affected site, and treatment modality (Table 3). The abnormal MPI result rate was significantly higher in subjects with pain duration at ≥15 months than those with pain duration at < 15 months (10.0% vs. 51.5%, *P* < 0.001). The mean FGR score was significantly lower in subjects with pain duration at ≥15 months (55.3 ± 2.5 vs. 42.0 ± 2.7, *P* < 0.001). Subjects with pain duration at ≥15 months also had higher FBR (52.1 ± 1.7 vs. 59.8 ± 2.4; *P* < 0.05), INC (50.6 ± 1.9 vs. 61.8 ± 2.3, *P* < 0.001), ANX (51.4 ± 2.0 vs. 64.7 ± 2.5, *P* < 0.001), DEP (48.9 ± 1.6 vs. 63.1 ± 2.3, *P* < 0.001), SOM (52.5 ± 2.5 vs. 66.3 ± 2.5, *P* < 0.001), PAR (51.1 ± 1.5 vs. 58.8 ± 2.0, *P* < 0.05), PDA (50.9 ± 1.6 vs. 63.7 ± 2.3, *P* < 0.001), and PDB (49.2 ± 1.6 vs. 61.6 ± 2.4, *P* < 0.001) scores (Table 4).

**Table 3:** Comparison of demographics and pain characteristics between the two complex regional pain syndrome (CRPS) groups according to pain duration

Variable	Pain duration < 15 mon group (*n* = 30)	Pain duration≥15 mon group (*n* = 33)	*P*
Age [year, median (range)]	19.3 (19.0–20.0)	19.3 (19.0–20.0)	0.086
Male [*n* (%)]	30 (100.0)	33 (100.0)	
Education [*n* (%)]			
College or more	20 (66.7)	20 (60.6)	0.617
High school or less	10 (33.3)	13 (39.4)	
Socioeconomic status [*n* (%)]			
High	5 (16.7)	3 (9.1)	0.385
Middle	17 (56.7)	16 (48.5)	
Low	8 (26.7)	14 (42.4)	
Parental coresidence status [*n* (%)]			
Both	27 (90.0)	27 (81.8)	0.710
One parent	3 (10.0)	5 (15.2)	
None	0 (0.0)	1 (3.0)	
mCSS (2–9)	4.53 ± 1.72	3.61 ± 1.32	0.019
NRS (0–10)	6.40 ± 1.65	6.12 ± 1.49	0.485
CRPS type [*n* (%)]			
I	23 (76.7)	26 (78.8)	0.841
II	7 (23.3)	7 (21.2)	
Affected site [*n* (%)]			
Upper extremity	10 (33.3)	8 (24.2)	0.209
Lower extremity	18 (60.0)	25 (75.8)	
Multiple	2 (6.7)	0 (0.0)	
Symptomatic presentation [*n* (%)]			
Allodynia	22 (73.3)	24 (72.7)	1
Hyperalgesia	22 (73.3)	21 (63.6)	0.410
Temperature asymmetry	17 (56.7)	17 (51.5)	0.680
Skin color asymmetry	21 (70.0)	16 (48.5)	0.083
Sweating asymmetry	17 (56.7)	11 (33.3)	0.062
Asymmetric edema	14 (46.7)	12 (36.4)	0.406
Trophic changes	8 (26.7)	4 (12.1)	0.142
Motor dysfunction	9 (30.0)	12 (36.4)	0.590
Decreased active range of motion	6 (20.0)	2 (6.1)	0.136
Treatment [*n* (%)]			
Physical or pharmacological therapy	8 (26.7)	13 (39.4)	0.540
Sympathetic nerve intervention	18 (60.0)	15 (45.5)	
Spinal cord stimulation	4 (13.3)	5 (15.2)	

*mCSS* Modified Complex regional pain syndrome Severity Score, *NRS* Numerical rating scale, *CRPS* Complex regional pain syndrome

**Table 4:** Comparison of Military Personality Inventory (MPI) results between the two complex regional pain syndrome (CRPS) groups according to pain duration

Variable	Pain duration < 15 mon group (*n* = 30)	Pain duration≥15 mon group (*n* = 33)	*P*
Abnormal MPI results [*n*(%)]	3 (10)	17 (51.5)	< 0.001**
Faking good response (*x* ± *s*)	55.3 ± 2.5	42.0 ± 2.7	< 0.001**
Faking bad response (*x* ± *s*)	52.1 ± 1.7	59.8 ± 2.4	0.012*
Infrequency (*x* ± *s*)	54.5 ± 1.9	60.6 ± 3.0	0.097
Inconsistency (*x* ± *s*)	50.6 ± 1.9	61.8 ± 2.3	< 0.001**
Anxiety (*x* ± *s*)	51.4 ± 2.0	64.7 ± 2.5	< 0.001**
Depression (*x* ± *s*)	48.9 ± 1.6	63.1 ± 2.3	< 0.001**
Somatization (*x* ± *s*)	52.5 ± 2.5	66.3 ± 2.5	< 0.001**
Schizophrenia (*x* ± *s*)	55.5 ± 1.8	61.1 ± 2.9	0.105
Paranoid (*x* ± *s*)	51.1 ± 1.5	58.8 ± 2.0	0.003*
Personality disorder cluster A (*x* ± *s*)	50.9 ± 1.6	63.7 ± 2.3	< 0.001**
Personality disorder cluster B (*x* ± *s*)	49.2 ± 1.6	61.6 ± 2.4	< 0.001**

*MPI* Military personality inventory. *P < 0.05. **P < 0.001

### Impact of pain severity and pain duration on the MPI scales

Univariate linear regression analysis indicated that mCSS is associated with SOM and PDA scores (*P* < 0.05). Pain duration was associated with FGR, INC, ANX, DEP, SOM, SCZ, PAR, PDA, and PDB scores (*P* < 0.05). In multivariate linear regression, pain duration was associated with FGR, INC, ANX, DEP, SOM, PAR, PDA, and PDB scores (*P* < 0.05 for all, Table 5).

**Table 5:** Univariable and multivariate linear regression analysis of pain severity and duration on the Military Personality Inventory (MPI) scales

Variable	Predictor	Univariate		Multivariate	
		Std. β	*P*	Std. β	*P*
Faking good response	mCSS	0.192	0.132	0.077	0.560
	NRS	0.134	0.294	0.093	0.466
	Pain duration	− 0.347	0.005*	−0.321	0.013†
Faking bad response	mCSS	0.003	0.982	0.055	0.693
	NRS	0.011	0.934	0.007	0.961
	Pain duration	0.180	0.157	0.196	0.146
Infrequency	mCSS	0.003	0.979	0.050	0.716
	NRS	0.092	0.472	0.094	0.476
	Pain duration	0.247	0.051	0.266	0.046
Inconsistency	mCSS	−0.137	0.285	−0.064	0.633
	NRS	0.012	0.924	0.050	0.699
	Pain duration	0.331	0.008*	0.317	0.016†
Anxiety	mCSS	−0.209	0.100	−0.086	0.495
	NRS	−0.068	0.596	−0.017	0.885
	Pain duration	0.454	< 0.001**	0.429	< 0.001†
Depression	mCSS	−0.240	0.058	−0.111	0.360
	NRS	−0.051	0.689	0.009	0.935
	Pain duration	0.506	< 0.001**	0.476	< 0.001†
Somatization	mCSS	−0.254	0.045*	−0.156	0.224
	NRS	−0.050	0.696	0.017	0.888
	Pain duration	0.415	< 0.001**	0.373	0.003†
Schizophrenia	mCSS	0.066	0.605	0.138	0.312
	NRS	0.050	0.698	0.027	0.834
	Pain duration	0.249	0.049*	0.289	0.030
Paranoid	mCSS	−0.193	0.130	−0.062	0.625
	NRS	− 0.104	0.419	− 0.061	0.619
	Pain duration	0.431	< 0.001**	0.410	0.001†
Personality disorder cluster A	mCSS	−0.263	0.037*	−0.111	0.355
	NRS	−0.131	0.305	−0.070	0.543
	Pain duration	0.513	< 0.001**	0.478	< 0.001†
Personality disorder cluster B	mCSS	−0.146	0.254	−0.035	0.784
	NRS	−0.013	0.917	0.022	0.856
	Pain duration	0.434	< 0.001**	0.426	< 0.001†

*Std. β* Standardized beta, *mCSS* Modified Complex regional pain syndrome Severity Score, *NRS* Numerical rating scale. *P < 0.05. **P < 0.001. †*P* < 0.016, adjusted significance level after Bonferroni corrections

## Discussion

A chronic state of illness or pain is often associated with reactive depression and anxiety as well as higher scores on many of the MMPI subscales [25, 26]. Additionally, among adolescents and children with CRPS, the risks of somatization, anxiety, and depression are high, and the duration of pain and depression has been shown to be associated with suicidal ideation [10, 11]. The results of the present study were consistent with previous studies in which the frequencies of anxiety, depression, and personality disorders were high among patients with chronic CRPS [27, 28].

We speculated that demographic characteristics, pain intensity, pain duration, clinical manifestations, and treatment modality invasiveness are associated with the psychopathology of adolescent CRPS. However, we failed to find associations between the psychopathology of adolescents with CRPS with demographic characteristics, the number of items in the IASP criteria, pain intensity, type of CRPS, the site of morbidity, or treatment modalities. Pain duration was the only factor that was significantly associated with psychopathology in our study.

Previous studies indicated that pain intensity is associated with psychopathology among patients with chronic pain [29, 30]. Such associations have also been observed among adult CRPS patients [31, 32]. We speculated that pain intensity would be associated with psychopathology in adolescent CRPS, but did not find evidence to support such an association. According to previous research by Logan et al. [33], children and adolescents from 7 to 18 years of age with CRPS had higher pain intensity ratings than other chronic pain groups. However, anxiety and depression were within the normal range in the CRPS group and did not differ from other chronic pain groups [33]. The pain duration among CRPS patients included in their study was 13.00 ± 2.9 months [33]. The fact that anxiety and depression did not differ from normative comparison data may be attributed to the relatively short pain duration.

Mesaroli et al. [34] retrospectively reviewed the charts of 59 CRPS patients with a mean age of 13.2 ± 2.6 years. The mean symptom duration was 35.7 ± 43.8 weeks. Their investigation using self-report questionnaires showed that anxiety, depression, and somatization were within normal ranges relative to age- and sex-matched peers [34]. However, through clinical diagnostic interviews, 39% of patients were diagnosed as having anxiety disorders, 12% had depressive disorders, 13% had somatic symptom disorders, and 37% had some type of past adverse events. There was a difference between the self-reporting and clinical interview methods.

The onset age of CRPS symptoms among our subjects ranged from 13 to 19 years, and the mean pain duration was 27.41 ± 2.67 months. The age and pain duration of the participants in the current study differed from that in previous studies by Logan et al. [33] and Mesaroli et al. [34], but the key results are generally consistent. As our study and both of these studies had retrospective designs, well-designed prospective studies are warranted.

Adolescent and pediatric CRPS have been associated with unique gender ratios [7, 8, 34]. CRPS is approximately 3 to 6 times more common among girls than boys [7, 8, 34]. The study sample investigated by Mesaroli et al. [34] was also predominantly composed of females (74.6%), and as previously mentioned, psychopathologic features were detected via clinical diagnostic interviews. Conversely, our study only included male subjects. Nonetheless, it is worth noting that the two previous studies had similar findings and that these results suggest that adolescent CRPS can manifest with psychopathology regardless of gender.

The stress process occurs as a bidirectional communication between the brain and the autonomic nervous system, the cardiovascular system, and the immune system through the neurological and endocrinological mechanisms responsible for cognition, perception, and behavior [35]. In “stressful” conditions, such as the experience of chronic pain, stress-related structural and functional plasticity can occur in corticolimbic structures, such as the hippocampus, amygdala, and the prefrontal cortex [35].

Recent studies have frequently reported that central neuroplasticity also progresses in CRPS [36–38]. Central neuroplasticity may occur as a form of change in gray matter or alterations in functional and white matter connectivity between brain regions [39]. These processes occur through mechanisms such as the unmasking or strengthening of silent or ineffective synapses, collateral sprouting, and the loss of gamma-aminobutyric acid (GABA) inhibition [39]. Several studies have suggested that gray matter volume is reduced in the insular cortex, nucleus accumbens, and prefrontal cortex of CRPS patients [36, 40]. Additionally, gray matter volume decreases with disease prolongation and progression, corresponding with structural and functional changes in the brain over time in CRPS [41].

Interestingly, previous studies have shown a reverse change, with increased gray matter volume and enhanced functional and structural connectivity after early treatment in pediatric and adolescent CRPS [42, 43]. However, a study by Linnman et al. [44] revealed that pain-induced alterations of functional connectivity in the brain could persistent even in a recovered state after functional recovery. Maladaptation of this plasticity can lead to the continuation and worsening of pain and is associated with psychopathology [35, 45].

Early diagnosis and treatment are known to be the major factors associated with successful outcomes for adolescent CRPS patients [46]. Active suspicion of adolescent CRPS and appropriate therapeutic interventions should be enacted promptly after diagnosis due to the potentially harmful effects of CRPS on adolescent development and future health. This will improve the quality of life by reducing pain and by reducing psychopathology by potentially shortening the pain duration. Therefore, there is a need to approach adolescent CRPS with a more time-limited concept when planning the treatment of patients in clinical practice.

Korea has a military draft policy. In young soldiers, delays in the diagnosis and treatment of trauma-associated pain that may occur in the military are likely to lead to CRPS. The newly developed psychopathology of soldiers can cause various problems, such as self-harm and harm to fellow soldiers. Therefore, the possibility of psychopathology is especially important for soldiers with chronic pain. However, psychological screening and treatment in the military remain insufficient compared with clinical practice outside the military. Within the military as well, the diagnosis and treatment of CRPS would have to be carried out thoroughly in a more time-limited concept. Psychological evaluations should be conducted to soldiers at appropriate intervals considering psychopathology that can change over time and treatment has to be followed even after the termination of pain. A multidisciplinary approach comprising psychological treatment should be initiated promptly when symptoms persist or worsen. Additionally, special attention should be paid when the soldier’s pain lasts longer than 15 months according to our result. In particular, it is worthwhile to consider early discharge as necessary after re-evaluation of psychopathology during the service period in the case of mandatory military service.

This study has several limitations. First, the present study was a retrospective cohort analysis based on self-reported assessments. Considering the difference between self-reported assessments and clinical presentations [34], the use of self-reported assessments in our study could have introduced some bias, although the reliability and validity of the MPI have been evaluated extensively [22, 23]. Second, because all of the participants were approximately the same age (19 years old) and were all males, caution should be exercised when generalizing the results to the general public, and the possibility of an association between a young age of onset and a distinctive psychopathological pattern cannot be overlooked. The third limitation is the use of an unvalidated modified version of the CSS. Last, psychopathology can be affected by the pain but also it can affect the pain experience vice versa [4, 6]. However, due to the nature of our data, we could not separately reveal the effect of the psychological characteristics on the experience of pain.

## Conclusions

Pain duration is the main factor affecting psychopathology among adolescents with CRPS. Psychopathologic features increased as the disease duration increased. A comprehensive understanding of time-dependent psychopathological manifestations could support a multimodal approach for detecting and managing adolescent CRPS.

## Data Availability

The datasets used and analyzed during the current study are available from the corresponding author on reasonable request.

## Abbreviations

ANX: Anxiety
AUC: Area under the curve
CI: Confidence interval
CRPS: Complex regional pain syndrome
CSS: Complex regional pain syndrome severity score
DEP: Depression
FGR: Faking good response
FBR: Faking bad response
GABA: Gamma-aminobutyric acid
IASP: International Association for the Study of Pain
INF: Infrequency
INC: Inconsistency
KIDA: Korea Institute for Defense Analysis
mCSS: Modified complex regional pain syndrome severity score
MMPI: Minnesota Multiphasic Personality Inventory
MPI: Military Personality Inventory
NRS: Numerical rating scale
OR: Odds ratio
PAR: Paranoid
PDA: Personality disorder cluster A
PDB: Personality disorder cluster B
ROC: Receiver operating characteristic
SOM: Somatization
SCZ: Schizophrenia
